# Clinical Implications of Cancer Related Inflammation and Depression: A Critical Review

**DOI:** 10.2174/1745017902117010287

**Published:** 2021-12-31

**Authors:** Daniel C. McFarland, Michelle Riba, Luigi Grassi

**Affiliations:** 1 Department of Medicine, Hofstra University, Northwell Health, Lenox Hill Hospital, New York, NY; 2 Department of Psychiatry, University of Michigan, Ann Arbor, MI; 3 Department of Psychiatry, University of Ferrara, Ferrara, Italy

**Keywords:** Depression, Inflammation, Cytokines, neurotransmitters, Neurocircuits, Clinical trials

## Abstract

Background: Neuropsychiatric symptoms are problematic in cancer settings. In addition to poor quality of life, depression is associated with worsened survival. Patients who develop depression that responds to treatment have the same cancer-related survival as those patients who never had depression. Although depression in patients with cancer is common, it is often unrecognized, untreated, or at best, undertreated. There remains untapped potential for underlying cancer-related biology associated with depression to help clinicians correctly identify depressed cancer patients and orchestrate appropriate treatments to address cancer-related depression. Biologically, inflammation has been most vigorously described in its association with depression in otherwise healthy patients and to a significant extent in patients with medical illness. This association is especially relevant to patients with cancer since so many aspects of cancer induce inflammation. In addition to cancer itself, its treatments (*e.g*., surgery, radiation, chemotherapy, and systemic therapies) and associated factors (*e.g*., smoking, obesity, aging) are all associated with increased inflammation that can drive immunological changes in the brain followed by depression. This critical review investigates the relationship between depression and cancer-related inflammation. It investigates several hypotheses that support these relationships in cancer patients. Special attention is given to the data that support certain inflammatory markers specific to both cancer and depression, the neurobiological mechanisms by which inflammation can impact neurotransmitters and neurocircuits in the brain, and the data addressing interventions that reduce inflammation and depression in cancer patients, and future directions.

## 1 INTRODUCTION

Decades of research have demonstrated high rates of depression in patients with cancer across various malignancies and settings (*e.g*., localized or metastatic cancer, throughout survivorship). Depression in the context of cancer can be devastating. It is associated with impaired quality of life, limited treatment adherence, and increased morbidity and mortality [1, 2]. Depression may stem from the multiple losses that patients with cancer experience such as work disruptions, decreased physical and cognitive ability, relationships strains, family structure disruption, and an altered sense of self. Yet, the psychological losses associated with cancer may only explain part of why patients with cancer develop depression so readily. Pancreatic cancer is known to induce depression long before physical symptoms appear, or an actual cancer diagnosis is made. This strange phenomenon led early clinicians and investigators to suspect a biological reason as at least partially driving depression in many cancer settings. Biological changes occur at the tumor and systemic levels and play a role in the development and progression of several neuropsychiatric symptoms including cancer-related fatigue, pain, sleep and cognitive impairment, and depression [1]. Cancer-related depression extends along a spectrum from mild to a debilitating psychiatric disorder occurring at one of the most physically vulnerable periods of a person’s life.

Despite the serious implications of having concurrent depressive symptoms alongside cancer and its high prevalence across cancer types, it is often not emphasized as a routine part of cancer care and remains unrecognized or inadequately treated, if addressed at all. Despite various efforts to institute distress or depression screening, or collaborative care models, the prevalence of depressive symptoms in the cancer setting remains high.

Inflammation has received increasing interest in psychiatry and oncology [3]. Neurotransmitter systems and neuropathways in the Central Nervous System (CNS) that are implicated in depression are also affected by inflammation induced by the presence of cancer [3]. This association is further validated because inflammation measured peripherally correlates with inflammation-induced changes in the CNS [2]. Given the multiple sources of inflammation and other biological changes that result directly from cancer and its treatment, patients with cancer may be especially vulnerable to many influences that inflammation has on the brain. Inflammation in the cancer context is exacerbated by surgery, chemotherapy, radiation, or certain types of immunotherapies in addition to direct tissue destruction or immune response from cancer. Cancer patients are vulnerable to opportunistic infections and changes in the microbiome (*e.g*., from antibiotics) also contribute to a chronic inflammatory response [4]. Additionally, psychological stressors stimulate chronic inflammation through the neuroendocrine system when stress is unremitting (*e.g*., sympathetic nervous system) [5]. Depressive symptoms associated with inflammation do not typically respond as well to conventional psychopharmacologic therapies [6].

## 2 METHODOLOGY

For the purposes of this review, we searched three databases, including PubMed, the Cochrane Library (Wiley), and PsycINFO (Ovid) to identify potentially relevant studies. The search had three primary categories, combined using the AND operator: (1) depression (2) inflammation, and (3) cancer up until December 2020. Studies were divided based on methodology, including human clinical studies (*e.g*., descriptive and interventional studies) and basic science, or lab-based studies. Reviews were also included in order to help organize previous studies.

### 2.1 Depression in Cancer

Depression is the most common psychiatric disorder in cancer patients and is elevated across cancer subtypes and is much more prevalent than in the general population [7]. It is also clear that rates of depression in cancer vary not only by cancer type but also by a number of additional factors including the measures used to assess depression, patient demographics, and cancer trajectory. Depressive symptoms are also greater with advanced disease stage and treatment-related symptom burden. Higher rates of depressive symptoms are typically found towards the end of life, and there is a significant overlap between physical symptom burden and depression. In general, 25-30% of patients experience some form of depressive disorder (percentages ranging from 4-60%) during cancer treatment [8]. Moreover, based on a meta-analysis of 70 studies across 14 countries, the pooled prevalence of a diagnosis of major depression was 16.3% in cancer patients [8].

Epidemiologic studies have revealed that all cancers except for localized thyroid cancer and non-melanoma skin cancer are associated with elevated rates of depression [8, 9]. Consistently, cancers of the lung and bronchus have the highest rates of depression and are also the most prevalent cancer types worldwide, highlighting the global burden of depression in cancer [10]. Several other cancer groups have high rates of depression, such as head and neck, pancreatic, gastric, and esophageal cancers but are individually less prevalent overall [11]. These cancers are also associated with significant physical morbidity and other specific psychological issues. For example, head and neck cancers are associated with loss of voice, disfigurement and impairment in significant bodily functions such as taste and smell that can severely limit patients’ ability to enjoy eating/drinking and ultimately social activities [12].

Overall, depressive symptoms exist on a continuum ranging from an appropriate response to medical illness to an adjustment disorder to minor depression and major depressive episodes. Unfortunately, depressive symptoms affect patients during the cancer trajectory when there may already be psychosocial issues related to family or relationship tensions, loss of autonomy, and changes in daily life due to cancer. At the same time when patients with cancer are being asked to manage a litany of tests, procedures, treatment schedules, and complex medical information, many are also experiencing symptoms of depression, especially at the beginning of their diagnosis, at a time when they most need to be present, involved, and engaged with family and loved ones. Whether co-morbid depression exists under a major or minor depressive category, significant functional impairment among patients with cancer and depression can affect the quality of multiple life domains, morbidity, and cancer-related mortality [1, 10]. In addition to suffering, reduced quality of life, progressive disability, ongoing depressive symptoms, or a co-morbid major depressive disorder are associated with poor adherence to recommended anti-cancer treatments, longer inpatient hospital stays, worsened overall survival (that can be ameliorated with remission of depression), and a desire for a hastened death [13-17].

Despite its obvious impact on the outcome, depression in cancer patients often goes undetected. Almost 60% of depressed patients are not diagnosed, with an even higher percentage in the geriatric population [18, 19]. [REMOVED HYPERLINK FIELD]Psychiatric disorders carry significant stigma even in the cancer setting. Patients do not want to ‘burden’ their oncologists by voicing psychosocial concerns. At the same time, oncology teams may be reluctant to ask or take the additional time necessary to inquire about depression. Furthermore, institutions vary in their readiness and approach to interdisciplinary workflows regarding distress and depression screening and referrals. There are also diagnostic challenges that may be responsible for the under-recognition of depression [20, 21]. Many patients, especially the elderly, focus on somatic rather than mood symptoms, and disentangling what symptoms are related to depression (*e.g*. poor appetite, sleep disturbances, fatigue, cognitive dysfunction) versus cancer and its treatment may be difficult if not impossible [22]. In a related fashion, the increased depression in cancer raises the question of its etiology and how much of the overlap is biological versus psychological [23]. Undeniably, there is a cacophony of psychological issues that patients and families with cancer experience throughout the cancer trajectory that are all related to the development and perpetuation of depression.

A related concern regarding depression in cancer is suicide. Rates of suicide in cancer are intimately tied to the time of cancer diagnosis despite commonly being quoted as twice the rate of the general population [24, 25]. Overall, patients with cancer are most likely to commit suicide in the first year of diagnosis but the relative risk is highest in the first few weeks (relative risk is 12.6) to three months (relative risk is 4.8) after diagnosis [25, 26]. The risk of suicide continues to be elevated up to at least 5 years after the cancer diagnosis while in certain cancers groups (*e.g*., bladder or renal cancer), the risk of suicide is continuously elevated over time [27].In addition to the length of time since diagnosis, the stage of disease at diagnosis is related to suicide risk as well [28]. Patients with more advanced disease at diagnosis are continuously at higher risk for suicide. Given the biological changes associated with suicide (*e.g*., lower serotonin levels in the central nervous system), the increased rate of suicide in cancer patients may also have a biological basis, possibly linked to inflammation, in addition to stemming from psychological suffering [29].

Thus, further examination of the relationship between depression and inflammation in cancer patients may help improve our understanding of the liabilities involved, while also providing clues as to which treatments may be especially relevant and ultimately efficacious for cancer patients. Toward that end, this study will review the many facets of the relationship among depression, inflammation, and cancer including the data that support increased rates of depression in cancer, the relationship between inflammation and cancer in non-cancer populations, the sources of inflammation in cancer patients, the relationship between cancer-specific inflammation and depression, the neurobiological mechanisms by which inflammation causes depression, and finally the impact of interventions and future directions.

#### 2.1.1 The Link Between Depression and Inflammation in Non-Cancer Populations

As indicated above, there are many factors that can contribute to depression in patients with cancer. Interestingly, however, one factor that is receiving increasing attention is inflammation. Many studies have demonstrated increased inflammatory markers in cancer patients with depression, and a body of data has demonstrated that inflammation can impact the brain to lead to depressive symptoms. Some of the earliest evidence of a relationship between inflammation and depression came from the study on the behavior of sick animals [30]. These behaviors, which occurred in the context of infection, included fatigue, listlessness and malaise, decreased appetite, reduced exploration, psychomotor slowing, social withdrawal, and behavioral inhibition. Later described as “sickness behavior,” these symptoms, which could be reproduced by the administration of inflammatory cytokines to laboratory animals and humans, were recognized as having significant overlap with depression [31]. Supporting the notion that inflammatory cytokines and the inflammatory response might be directly linked to depression in humans, in 1993, it was reported that acute-phase proteins, important components of the inflammatory response, increased in patients with major depression [32]. Subsequently, a multitude of clinical studies have found significant associations between numerous markers of inflammation and depression [3]. Indeed, meta-analyses of this literature have concluded that peripheral blood levels of the inflammatory cytokines interleukin (IL)-1beta, IL-6, and tumor necrosis factor (TNF) and the acute phase reactant c-reactive protein (CRP) are the most reliable biomarkers of inflammation in patients with depression [33, 34]. It should be noted that many of these inflammatory markers are also used as prognostic markers in the cancer setting and have other clinically relevant roles. Depression with evidence of increased inflammation is characterized by greater persistence and severity, later age of onset, poor response to treatment, and reduced motivation [6, 35-37]. In addition to the cross-sectional association of increased inflammatory markers with depression, there are ample data supporting a longitudinal relationship between inflammation and depression as evidenced by systematic review and meta-analyses that have reported higher concentrations of CRP and IL-6 in association with increased risk for the development of a depressive disorder [38]. Further compelling clinical evidence of a link between depression and inflammation is the presence of depressive symptoms following the administration of inflammatory cytokines [3]. For example, administration of the inflammatory cytokine interferon (IFN)-alpha for the treatment of malignant melanoma or renal cell carcinoma is associated with clinically significant depression in 30-50% of patients [39]. Similar depressive symptoms have been found following the administration of other inflammatory stimuli, including low dose endotoxin and typhoid vaccination to healthy volunteers [3]. Finally, data indicate that blockade of inflammatory cytokines can reduce depressive symptoms, especially in patients with autoimmune and inflammatory disorders receiving anti-cytokine therapies [40].

#### 2.1.2 Sources of Inflammation in Cancer

There are many sources of inflammation in patients with cancer. Not only does cancer itself contribute to increased inflammation, but also many of the treatments used to treat cancer, including surgery, chemotherapy, and radiation, are associated with significant tissue damage and destruction that can activate the inflammatory response. For example, inflammatory cells in the tumor environment produce high levels of IL-6 and other inflammatory cytokines as well as disruptions in T cell populations [41]. IL-6 promotes angiogenesis, invasion, attachment, and generation of tumor-associated macrophages [42]. Elevations of IL-6, along with other inflammatory markers are also associated with decreased time to tumor recurrence and shorter survival time in patients with ovarian cancers [43]. Of relevance to depression, increased IL-6 as a result of an implanted tumor has also been associated with depressive-like behavior in laboratory animals, indicating the possibility that the presenting symptom in patients with cancer may be depression [44]. Data also suggest that treatments such as chemotherapy might lead to persisting changes in inflammatory setpoints through epigenetic effects of genes related to inflammatory signaling pathways. Indeed, chemotherapy was shown to decrease methylation in CpG(cystine, guanine) sites associated with several genes, including *TMEM49* (Transmembrane Protein-49) in peripheral blood immune cells that were directly correlated with peripheral blood concentrations of IL-6 and soluble TNF Receptor 2 (sTNFR2) [45]. Increasing attention has been paid to other sources of inflammation in the context of cancer, including the use of immunotherapeutic treatments such as checkpoint inhibitors, which have been associated with an increased incidence of autoimmune disorders [46]. In addition, disruptions in the microbiome as a result of cancer and its treatment may contribute to alterations in the inflammatory response [4]. Finally, the multitude of psychosocial stressors associated with a cancer diagnosis as well as early life stress may contribute to increased inflammation through activation of the sympathetic nervous system and the hypothalamic-pituitary-adrenal response [5, 47, 48].

#### 2.1.3 Inflammation and Depression in Cancer Patients

Given the relationship of inflammation with depression and the many sources of inflammation in cancer patients, many studies have examined the relationship between inflammation and depression in cancer patients. A cursory, non-systematic review of studies found a total of 38 studies (including 8 intervention studies) meeting the criteria of 1) depression, 2) cancer setting, and 3) evaluation of any marker of inflammation [23]. The majority of these studies, *i.e*., 35 out of 38, found that inflammatory markers were higher in cancer patients with depression, although the results may or may not have reached statistical significance. Some studies did not assess depression or inflammation as a predetermined outcome, and of these, not all of them were the primary outcome of interest. The cancer diagnosis represented in the papers examined was largely breast cancer (14 studies), followed by heterogeneous cancer diagnoses, including lung cancer (5 studies), renal cell cancer (2 studies), colorectal cancer (2 studies), and hematologic malignancy, hepatocellular carcinoma, melanoma, ovarian, pancreatic, and testicular (all 1 study each). Of the inflammatory markers, inflammatory cytokines were most commonly evaluated (31 studies) with IL-6 and TNF being the most commonly measured. The next most common inflammatory marker was the positive acute phase reactant CRP (9 studies) with additional studies examining albumin (negative acute phase reactant) and the erythrocyte sedimentation rate (ESR). Depression measures were most consistently represented by the Hospital Anxiety and Depression Scale (HADS) (10 studies), Hamilton Depression Rating Scale (HAM-D) (8 studies), CES-D (9 studies), SCID (5 studies), Patient Health Questionnaire (PHQ-9) (2 studies), and Profile of Mood States (POMS) (2 studies), along with 9 other scales that were only represented in one study. These studies all revealed an association between depression and inflammation. It should be noted that historically, studies in this area of psychoneuroimmunology have suffered from methodological flaws (*e.g*., underpowering for hypothesis testing, insufficient attention to the timing of cytokine collection). While studies are heterogeneous in terms of cancer setting, sample size, and measure of inflammation and depression, they provide support for the notion that inflammation and depression have been reproducibly associated across multiple cancer types and settings. It should be noted, however, that not all cancer patients with depression have increased inflammation [49]. The percentage of patients with increased inflammation (defined as a CRP>3mg/L) has not been well studied in patients with cancer, but in otherwise healthy depressed subjects, the percentage is ~28% rising to almost 50% in treatment-resistant depressed subjects [50, 51].

#### 2.1.4 How does Inflammation Impact the Brain and Lead to Depression?

Based on studies on laboratory animals and humans as well as cell-culture experiments, a great deal of information has been amassed that provides important clues as to how inflammation affects the brain to influence behaviors including symptoms of depression [3]. Inflammatory cytokines have been shown to affect virtually every aspect of the metabolism of monoamines, including serotonin, norepinephrine, and dopamine [3, 47].Monoamines play a critical role in the regulation of mood and are the target of conventional antidepressant medications. Effects of inflammation include reduction in monoamine synthesis and release as well as an increase in reuptake [47]. For example, through the generation of reactive oxygen and nitrogen species, inflammatory cytokines have been found to decrease the availability of tetrahydrobiopterin (BH4), which is a critical enzyme cofactor in the synthesis of all of the monoamines. In addition, inflammatory cytokines especially interferon (IFN)-gamma and TNF can activate the enzyme indoleamine 2,3dioxygenase (IDO) that converts tryptophan (the amino acid precursor of serotonin) into kynurenine [31, 47]. Kynurenine can in turn be converted into kynurenic acid and quinolinic acid in the brain, where they both have neuroactive properties that may contribute to depression [31]. Studies involving humans and nonhuman primates have also demonstrated that the inflammatory cytokine IFN-α leads to decreased release of dopamine [52]. Moreover, both IL-1β and TNF-α have been shown to increase the expression and function of serotonin and norepinephrine reuptake pumps through stimulation of p38 mitogen-activated protein kinase [47]. In addition to the effects of inflammation on monoamines metabolism, inflammatory cytokines have also been shown to have profound effects on glutamate, leading to an increased release and decreased reuptake of glutamate from astrocytes [53]. Increased glutamate has been found in the basal ganglia and dorsal anterior cingulate cortex in patients treated with IFN-alpha as well as in depressed patients with increased inflammation as indexed by CRP [53]. Increased glutamate is believed to lead to extrasynaptic spillover, resulting in the binding of glutamate to extrasynaptic glutamate (N-methyl-D-aspartate) receptors, which can decrease essential growth factors such as brain-derived neurotrophic factor (BDNF) and contribute to excitotoxicity [53]. Inflammatory cytokines have also been shown to have direct effects on BDNF and ultimately neurogenesis, contributing to alterations in neural plasticity [3, 47].The effects on glutamate metabolism have been associated with decreased regional homogeneity (reflecting chaotic neuronal activity) in brain regions associated with depressive symptoms including anhedonia and psychomotor retardation [54]. Consistent with the effects of inflammatory cytokines on neurotransmitter systems and neural plasticity, neuroimaging studies have demonstrated reproducible effects of inflammatory stimuli on neurocircuits involved in motivation and motor activity as well as anxiety, arousal, and alarm [3]. Chronic administration of IFN-alpha and acute administration of endotoxin or typhoid vaccination have all been shown to decrease activation of reward regions in the brain including the ventral striatum [3, 52, 55]. Based on studies involving laboratory animals, these effects appear to be related to the impact of inflammatory cytokines on dopamine metabolism in the ventral striatum including the nucleus accumbens [55]. Interestingly, in a study on mice, stress-induced inflammation led to a selective increase in the blood-brain barrier in the area of the nucleus accumbens, only in animals that developed depressive-like behavior in response to stress [55]. Inflammation-induced effects on the ventral striatum have been associated with decreased effort-based motivation, while sensitivity to reward appears to remain intact [55]. Of note, consistent with studies administering inflammatory stimuli, increased endogenous inflammation in depressed patients as indexed by CRP has been associated with decreased functional connectivity within reward circuitry including connectivity between the ventral striatum and the ventromedial prefrontal cortex [37]. These connectivity changes were in turn correlated with anhedonia [37]. Administration of inflammatory stimuli has also been associated with activation of neurocircuitry related to anxiety including the dorsal anterior cingulate cortex, insula, hippocampus, and amygdala [3]. In addition, in patients with depression, increased inflammation was associated with decreased functional connectivity between the ventromedial prefrontal cortex (vmPFC) and the amygdala that was especially apparent in individuals with co-morbid anxiety disorders [56]. Taken together, these data provide strong support for the notion that increased inflammation can affect the fundamental pathways that regulate behavior with reproducible effects on brain regions that are associated with depressive behaviors. Effects on motivation and motor activity as well as anxiety and alarm appear to be related in part to the impact of inflammation on dopamine metabolism and reward-related neurocircuitry.

#### 2.1.5 Interventional Studies on Depression and Inflammation

Several interventional studies have been conducted in cancer settings that have addressed the relationship between depression and inflammation. For example, in a small study, the anti-depressant effect of the anti-inflammatory COX-2 inhibitor celecoxib was examined in depressed patients with colorectal cancer receiving chemotherapy. Compared to placebo, celecoxib-treated patients exhibited a greater decrease in depressive symptoms at 4 and 6 weeks [57]. This proof-of-concept study has particular relevance in the colorectal cancer setting where anti-inflammatory medications may also confer a cancer-related survival benefit. Non-pharmacologic interventions for depression have also been explored with the idea that treating depressive symptoms may lead to a reduction in inflammatory markers in cancer patients. Indeed, mindfulness-based stress reduction (MBSR) was found to reduce both depressive symptoms and inflammatory cytokines in patients with metastatic breast cancer [58]. MBSR also demonstrated a similar effect on depression and inflammatory cytokines in patients with localized breast cancer [59]. A group therapy intervention spanning 12 months in newly diagnosed breast cancer patients was also found to decrease depression and markers of inflammation, and the intervention’s effect on inflammation was shown to be mediated by the reduced depressive symptoms [60]. Finally, an intervention that targeted the patient-caregiver dyad in a web-based collaborative trial of 261 patients and 179 caregivers led to reduced depression, pain, fatigue, along with reductions in pro-inflammatory cytokines (IL-6, IL-1β) [61]. Taken together, these data suggest that non-pharmacologic interventions that reduce distress may treat both depression and inflammation, although, with the limited data available, the directionality of these effects remains unclear (*e.g*. can inflammation reduce by decreasing depression or can depression reduce by decreasing stress-related inflammatory pathways?).

## CONCLUSION AND FUTURE DIRECTIONS: ADDRESSING DEPRESSION AND INFLAMMATION IN CANCER

Given the growing literature on the impact of inflammation on the brain and the many sources of inflammation in cancer patients, there is increasing interest in applying what is known about how inflammation can influence the brain and behavior to treat depression in cancer patients. In general, two strategies might be considered: 1) targeting inflammation itself or 2) targeting the downstream effects of inflammation on the brain.

### Targeting Inflammation

There are many strategies that might be employed to reduce inflammation to treat depression. As noted previously, a number of studies have shown that pharmacologic anti-cytokine therapies have efficacy in reducing depression in patients with autoimmune and inflammatory disorders as well as in otherwise healthy depressed subjects [40, 62]. The effects of anti-cytokine therapies in otherwise healthy depressed individuals seem to be especially effective on motivational deficits and anhedonia [51]. Other anti-inflammatory medications, including celecoxib and aspirin, which block cyclooxygenase 2 and prostaglandin synthesis, and minocycline which reduces activation of microglia in the brain, have shown antidepressant activity, although the literature in this area remains limited at this time [63]. Diet and exercise have also been shown to reduce the inflammatory responses with the Mediterranean diet exhibiting reliable anti-inflammatory properties [63]. As indicated above, meditation and yoga decrease inflammatory markers, an effect believed to be related in part to an augmentation of parasympathetic nervous system function, which through cholinergic signaling exhibits anti-inflammatory effects [63]. Other considerations include the reduction of oxidative stress through diet and exercise as well as environmental exposures, including pollutants and radiation. Given the data supporting the role of inflammation (as well as oxidative stress) in cancer development and progression, the potential promise of therapeutic strategies targeting inflammation to treat depression is that they may impact cancer outcomes as well.

### Targeting the Downstream Effects of Inflammation on the Brain

Based on the rich literature detailing the effects of inflammation on brain neurotransmitter systems and neurocircuits, there are many potential opportunities for the development of pharmacologic treatment strategies to mitigate the effects of inflammation on the brain. Probably the readiest for primetime is the use of medications that target the effects of inflammation on dopamine and glutamate [63]. As noted above, inflammation can impact the synthesis and release of dopamine, and drugs that facilitate dopamine availability have been shown to reverse the effects of inflammation on depressive behaviors in both laboratory animals and humans. These medications include the dopamine reuptake inhibitor bupropion and the stimulant methylphenidate [63].In addition, data indicate that the glutamate antagonist ketamine is more effective in patients with increased inflammation, suggesting that blocking the glutamate receptors with drugs, like ketamine or memantine, may mitigate the effects of inflammatory cytokines on glutamate release and reuptake by astrocytes and microglia. Another strategy that warrants consideration is the disruption of the kynurenine pathway by blocking IDO with 1-methyltryptophan or by inhibiting the transport of kynurenine into the brain with leucine, which competes with kynurenine for the large neutral amino acid transporter [64]. Given the impact of inflammation on specific neurocircuits in the brain, it is also possible to target neurocircuits through neuromodulation using treatments such as transcranial magnetic stimulation and electroconvulsive therapy (ECT). For example, increased IL-6 predicted an increased likelihood of response to ECT [65]. Taken together, the emerging data suggest that the development of treatments that target the downstream effects of inflammation on the brain may be worthy of future examination in clinical trials. Of note, clinical trial designs should include match/mismatch designs including cancer patients with low and high inflammation [63]. Drugs targeting inflammation or its downstream effects on the brain should be efficacious in patients with high inflammation but not low inflammation.

Finally, the impact of inflammation on the brain leading to depression not only has treatment implications but also may contribute to the identification of cancer patients at high risk for depression. Given the devastating effects of depression on quality of life and cancer outcomes, clinicians should have a high suspicion for depression in patients undergoing treatments for cancer that lead to increased inflammation, especially in patients who may have other potential psychosocial and genetic risk factors. Tests for inflammation, including simple blood tests such as CRP, can be used and may help serve as a marker of the biological risk for depression.

## LIST OF ABBREVIATIONS

BDNF: = Brain-Derived Neurotrophic Factor
BH4: = Tetrahydrobiopterin
CES-D: = Center for Epidemiologic Studies Depression Scale
CNS: = Central Nervous System
COX-2: = Cyclooxygenase 2
CpG sites: = Cysteine/Guanine nucleotides
CRP: = C-Reactive Protein
ECT: = Electroconvulsive Therapy
ESR: = Erythrocyte Sedimentation Rate
HADS: = Hospital Anxiety Depression Scale
HAM-D: = Hamilton Depression Rating Scale
IL-1β: = Interleukin 1β
IL-6: = Interleukin 6
INF-γ: = Interferon gamma
MBSR: = Mindfulness-Based Stress Reduction
PHQ-9: = Patient Health Questionnaire 9
POMS: = Profile of Mood States
SCID: = Structural Clinical Interview for DSM-5
*TNEM49*: = Transmembrane Protein 49 (gene)
TNF-α: = Tumor Necrosis Factor α
vmPFC: = Ventromedial Prefrontal Cortex
